# 3D Imaging Millimeter Wave Circular Synthetic Aperture Radar

**DOI:** 10.3390/s17061419

**Published:** 2017-06-17

**Authors:** Renyuan Zhang, Siyang Cao

**Affiliations:** Department of Electrical and Computer Engineering, University of Arizona, Tucson, AZ 85721, USA; ryzhang@email.arizona.edu

**Keywords:** radar 3D imaging, synthetic aperture radar, millimeter wave radar, remote sensing, compressed sensing, inverse Radon transform, portable, circular SAR

## Abstract

In this paper, a new millimeter wave 3D imaging radar is proposed. The user just needs to move the radar along a circular track, and high resolution 3D imaging can be generated. The proposed radar uses the movement of itself to synthesize a large aperture in both the azimuth and elevation directions. It can utilize inverse Radon transform to resolve 3D imaging. To improve the sensing result, the compressed sensing approach is further investigated. The simulation and experimental result further illustrated the design. Because a single transceiver circuit is needed, a light, affordable and high resolution 3D mmWave imaging radar is illustrated in the paper.

## 1. Introduction

3D imaging radar becomes feasible in the field of remote sensing due to the advancement in solid state microwave circuits and the digital signal processor [1]. However, large antenna synthesization is usually performed by airborne radar to interrogate the terrain [2] and is not suitable for civilian usage. Considering a wide usage of millimeter wave radar for automobile, security and surveillance, we are proposing a new imaging technique [3,4] combining synthetic aperture radar (SAR) and millimeter wave radar. The proposed millimeter wave circular synthetic aperture radar (MMWCSAR) conducts a circular trajectory to synthesize a large aperture for achieving 3D imaging. MMWCSAR has four key aspects: high resolution, working on diversity conditions, portable and low-cost.

For high resolution, millimeter wave radar can support hundreds of megahertz for range detection. For example, automotive radar is working on millimeter wave. It can sweep hundreds of megahertz to distinguish cars and even pedestrians [5]. In addition, automotive radar has been applied on smart vehicles for 2D terrain mapping [6], collision detection, parking assistance and the blind spot indicator [7]. However, the current automotive radar uses traditional strip-map SAR imaging to generate a 2D terrain mapping. The proposed MMWCSAR aims at generating 3D imaging. It can be further applied to automotive radar for better understanding of the environment.

For working on diversity conditions, we compare millimeter wave radar with optical technologies. Despite that optical imaging techniques have better resolutions due to the high center frequency, radar imaging has numerous advantages over traditional optical imaging techniques like camera and LiDAR. It is underlined in [8] that radar has superior working capacity in any weather condition, including rain, snow and fog. The complex roadway environment for automobiles requires uninterrupted remote sensors performing consistently in inclement weather. The millimeter wave radar is capable of acquiring and tracking all obstacles in its field of view (FOV) under all weather conditions [8]. MMWCSAR is an innovative imaging device capable of working in diverse conditions compared to traditional optical sensors.

For portable and low-cost features, we compare with traditional millimeter wave imaging techniques, such as security surveillance system [9,10], concealed weapon detection [11,12,13] and millimeter wave cameras [6,14]. Most of these traditional applications, including the millimeter wave scanner, which deploys many transceivers, are bulky and high-cost system designs. On the contrary, MMWCSAR adopts a monostatic radar, which can minimize its size and cost. It ensures that MMWCSAR can provide three-dimensional microwave imaging for security, indoor surveillance and automotive target detection in a wearable and inexpensive manner.

MMWCSAR requires the motion of radar in a circular track other than a straight track as traditional strip-map SAR techniques. Our study found that the movement between radar and targets produces the projections of objects to different movement directions. One can therefore obtain a range projection angle datacube from the circular movements. From the datacube, by applying inverse Radon transform (IRT) [15,16], the datacube can be converted to the FOV figure. The principles are similar to computed tomography [15,17,18,19,20]. Jia et al. [21] presented a 2D imaging algorithm for circular SAR. The motion of the SAR is along a circular trajectory. The sub-spectrums of different angles are matched filtered and summed in the Fourier domain to obtain a 2D Fourier spectrum of the imaging. By space-invariant matched filtering and 2D inverse Fourier transform (2D-IFFT), the trajectory deviation is eliminated, and the final aperture view is presented. Additionally, Bao et al. [22] proposed a multi-circular SAR approach. Their multi-circular SAR samples on different elevation levels. Different circular rotation angles and down-looking angles are recorded according to the range data. Consequently, their signal processing procedure results in reconstruction of a 3D image from multi-circular SAR. In our approach, we can use hand swinging to obtain a full-circular projection. The circular trajectory plane is parallel to the range bin plane. The range calibration is also implemented before the IRT in order to reduce target mismatch on the volume FOV image.

Traditional spotlight SAR works on S-band or X-band. It has a low carrier frequency and uses the range information to perform inverse radon transform. In the paper, we are investigating the W-band 3D imaging technique. We found that as the carrier frequency increases to the W-band, the synthetic aperture becomes much smaller and allows the radar to use Doppler angle data to perform inverse Radon transform. The advantages of the proposed system are multifold: First, the high carrier frequency makes the radar very small. Second, the lesser number of transceiver elements can lower the cost of the radar. Third, further study found that a high resolution result can be obtained with less samples if compressed sensing (CS) is applied.

MMWCSAR tested with hand swinging is not accurate for high resolution imaging. In order to improve the sensing results, sensing time and improve the rotating accuracy, additional signal processing procedure of CS can be applied in our MMWCSAR system. CS has been introduced in [23,24]. Many large data size applications behaving as sparse targets have been using CS for data reduction and restoration, e.g., single-pixel imaging [25] and magnetic resonance imaging (MRI) [26,27]. It has advantages for situations when sampling is expensive, slow or difficult [28]. The CS method applied in radar signal processing can sample fewer signals for the sensors, while keeping the same quality of the generated imaging. With reducing the size of samples for the portable radar device like MMWCSAR, a fast data acquisition can be achieved by the CS method. Meanwhile, the sensing results can be improved.

Radar signal processing with CS is addressed in [2,21,22,29,30,31,32]. In [29], Ender gave a full analysis on applying the CS to radar pulse compression. Sevimli [30] introduced range-Doppler compressed sensing and optimization comparison of different reconstruction algorithms. In addition to the CS used in range-Doppler response, our method of applying CS to radar signal processing is innovative. It allows not only the pulse-Doppler compressed sensing, but also the slow-time-angle compressed sensing. Recent work on CS applied to SAR systems is drawing researchers’ attention. For instance, Bao et al. [22] produced the 3D multi-circular SAR image using a “2D + 1D” mode, i.e., 2D focused SAR images are followed by 1D profile estimation of the elevation direction. With CS applied to the 2D ground plane image and 1D profile of the elevation dimension, the 3D figure can be reproduced. CS theory is also applicable in our MMWCSAR system. First, CS is applied to 2D slow-time-angle data to form a 2D FOV image on a single range bin. The volume FOV figure can be reconstructed and analyzed by applying the 1D range profile to 2D FOV images of different range bins. Second, to achieve the CS in radar signal processing, the sparsity and incoherence properties are discussed. In addition, 2D transformation from the slow-time-angle to the azimuth-elevation representation matrix is discussed. Besides, we also introduce how to choose the sensing matrix, so that the CS algorithm can be realized. Finally, to further improve the performance of the MMWCSAR system, we focus on decreasing the data acquisition time, improving imaging results and reducing errors caused by humans with the CS algorithm applied in the experiment.

The structure of this paper is as follows. In Section 2, the MMWCSAR system configuration, parameters, data acquisition, resolution and its constraints of choosing MMWCSAR system parameters are introduced. In Section 3, range calibration and radar imaging processing by using IRT to reconstruct the volume FOV image are presented. In Section 4, we propose the CS for MMWCSAR algorithm. The corresponding simulation, as well as experimental results are shown in Section 5. In Section 6, we discussed the results from the simulation and experiment. In Section 7, we conclude the paper.

## 2. MMWCSAR System

The relative movement between a radar and targets can be used to detect and locate targets. Examples include SAR [2,33], inverse SAR (ISAR) [34,35,36,37], moving target indicator [7], etc. To introduce the relative movement between the proposed radar and targets, the placement of the MMWCSAR system is presented in Section 2.1. The parameters for the monostatic radar are introduced in Section 2.2. In Section 2.3, data acquisition is shown. The resolution of the MMWCSAR system is presented in Section 2.4. The constraints of MMWCSAR parameters are studied in Section 2.5.

### 2.1. MMWCSAR Configuration

**Figure 1:**
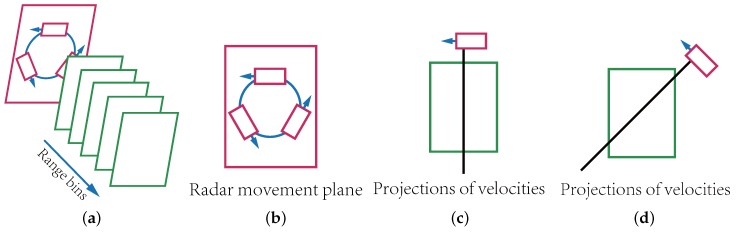
Uses rotation radar to resolve 3D imaging. (**a**) Schematic view of resolving range using range bins; (**b**) the swinging within the rotation plane of the radar generates velocities in different directions; (**c**,**d**) each range bin is then projected into different velocity directions while data are collected.

The proposed 3D imaging MMWCSAR system uses a single transceiver element to acquire data from targets by emulating multiple transceivers through movement of the single transceiver. To simplify the movement of MMWCSAR, we assume that MMWCSAR moves along a circular track. The plane of the circular track is perpendicular to the range bin axis (in Figure 1a). The radar moving along the circular track keeps a constant speed, but the direction changes over time (in Figure 1b).

As the radar is moving inside the plane perpendicular to the range bin axis, targets detected remain stationary within its range bin while sampling. The movement of radar produces relative speeds of detecting targets. For each separate range bin, detected targets have different relative velocities depending on their azimuth and elevation locations. As the radar moves to different directions, targets within a single range bin project to different directions. If the target is at the center of its range bin, the relative velocity is zero no matter the radar’s movement direction. For targets away from the center, the relative velocities vary according to the radar’s movement directions. Figure 1c,d shows that targets within a range bin are projected onto different radar moving directions. Consequently, relative movement produces targets’ Doppler data, which can be used to distinguish targets within the same range bin.

**Figure 2:**
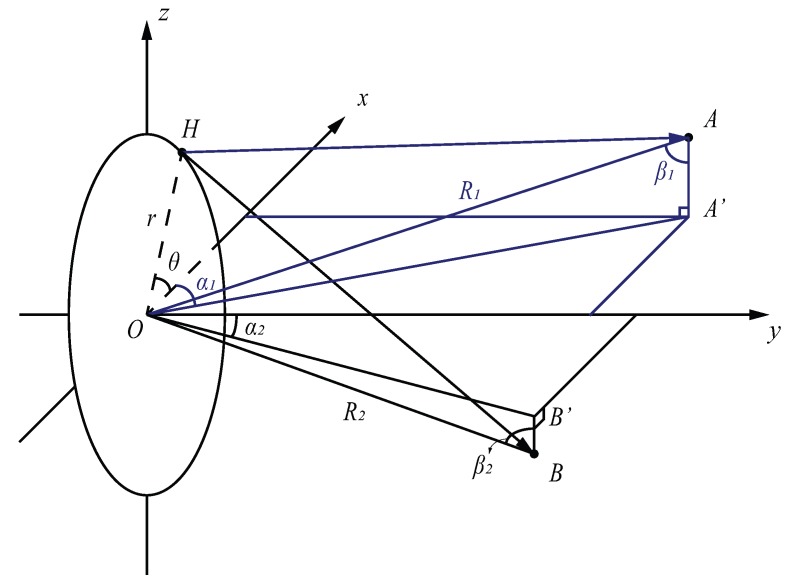
Geometry of monostatic radar remote sensing targets (the positive *y* axis is the boresight direction).

As we can see from Figure 2, the geometry of MMWCSAR is presented. *H* represents a monostatic radar. For different rotation positions, the range-azimuth-elevation of the radar can be presented in Cartesian coordinates as $\left(H_{x},\;0,\;H_{z}\right)$: $H_{x}=r\cos\theta$ and $H_{z}=r\sin\theta$, where *r* denotes the rotation radius of the MMWCSAR system, and θ is the rotation angle at which the MMWCSAR takes a frame of 2D range-Doppler data. θ is related to the angular velocity ω and acquisition time stamp *t*, i.e.,

(1) $$\theta =\frac{2\pi t}{\omega }\;.$$

*A*, *B* are point targets ahead of the radar with different positions, and their Cartesian representation are $\left(A_{x},\;A_{y},\;A_{z}\right)$ and $\left(B_{x},\;B_{y},\;B_{z}\right)$. As the spherical coordinates are used for signal processing, *A* and *B* have spherical coordinates’ profiles of $\left(R_{1},\;\alpha_{1},\;\beta_{1}\right)$ and $\left(R_{2},\;\alpha_{2},\;\beta_{2}\right)$. The radar-target vector components of $\vec{HA}$ and $\vec{HB}$ relative to radar in Cartesian coordinates are:

(2) $$\vec{HA}=\left(R_{1}\sin\beta_{1}\cos\alpha_{1}-r\cos\theta ,R_{1}\sin\beta_{1}\sin\alpha_{1},R_{1}\cos\beta_{1}-r\sin\theta \right)$$

and:

(3) $$\vec{HB}=\left(R_{2}\sin\beta_{2}\cos\alpha_{2}-r\cos\theta ,R_{2}\sin\beta_{2}\sin\alpha_{2},R_{2}\cos\beta_{2}-r\sin\theta \right)\;,$$

respectively. Equations (2) and (3) provide a method to describe targets in terms of range, azimuth angle and elevation angle instead of range, azimuth location and elevation location. Targets’ 3D location profiles are independent of the radar movement as long as applying the range calibration of the displacement of the origin (radar rotation center) to the radar. The projections are projected onto each range bin and are associated with rotation angle θ. Therefore, the MMWCSAR addresses a unique approach of the radar remote sensing problem.

**Figure 3:**
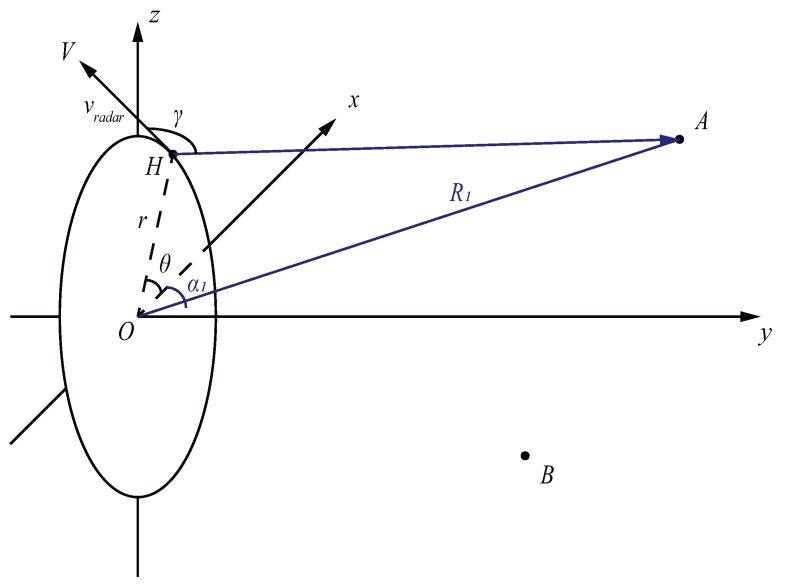
Geometry of monostatic radar velocity projections (the positive *y* axis is the boresight direction).

The moving direction can be recorded by angle θ over time. The geometry of obtaining the projection angle γ can be seen from Figure 3. Doppler velocities of targets are the projections onto the radar rotation plane. γ can be represented using the displacement vector $\vec{HA}$ and the velocity vector $\vec{HV}$:

(4) $$\gamma =\arccos\frac{\vec{HA}\cdot \vec{HV}}{\left|\vec{HA}\right|\left|\vec{HV}\right|}\;.$$

From geometry, we know that:

(5) $$\begin{array}{cc} \vec{HA}\cdot \vec{HV} & =\left(\vec{OA}-\vec{OH}\right)\cdot \vec{HV}\\ & =\vec{OA}\cdot \vec{HV}-\vec{OH}\cdot \vec{HV}\\ & =\vec{OA}\cdot \vec{HV}\;. \end{array}$$

$\left|\vec{HA}\right|$ is the actual range of radar and target, $R_{revised}$. The range difference between the assumed range $R_{assumed}=\left|\vec{OA}\right|$ and $R_{revised}$ will be discussed in range calibration Section 3.1. Thus, the Equation (4) can be simplified as:

(6) $$\gamma =\arccos\frac{\vec{OA}\cdot \vec{HV}}{R_{revised}\left|v_{radar}\right|}\;.$$

$\left|v_{radar}\right|$ is the magnitude of the velocity of the radar:

(7) $$\left|v_{radar}\right|=\frac{2\pi r}{\omega }\;.$$

All vectors are derived from coordinates calculations.

### 2.2. MMWCSAR Parameters

As introduced above, to build a light and low-cost imaging radar, we use a monostatic radar with a single transceiver element.

In our setups, the monostatic radar is transmitting linear frequency modulated (LFM) pulse waveforms and operating in range-Doppler mode. The intermediate frequency (IF) of the radar is defined as center frequency, $f_{c}$. The bandwidth (BW) of the radar determines the range resolution. In our millimeter wave design, we use a wide bandwidth chirp. Sampling frequency, $f_{s}$, and pulse chirp duration, $T_{P}$, define the number of range bins, $N_{R}$. Pulse repetition interval (PRI) generally determines the blind speed and hence the unambiguous Doppler frequency, $f_{d}$ [38]; thus limiting the swinging angular velocity, ω, in our MMWCSAR system. The number of Doppler bins, $N_{D}$, defines the velocity resolution and curbs the scanning frames, $N_{Ch}$. The number of frames collected is fundamental to the final FOV image.

### 2.3. Data Acquisition

The radar transmission signal s(t) is the LFM signal. The observation of radar signal for a single scatterer after de-ramping is:

(8) $$r\left(t\right)=k\exp\left[j2\pi \left(\frac{2R}{c}\frac{\left(BW\right)}{T_{P}}+\frac{2vf_{c}}{c}\right)t\right]+n\left(t\right)\;.$$

where *k* is the reflective amplitude related to the target’s radar cross-section (RCS), n(t) is the white noise, *R* is the range of the target, *v* is the velocity of the target and *c* is the speed of electromagnetic wave. Two terms of the frequency component in the exponential function are fast-time and slow-time samples. These samples are the frequency difference of range and Doppler, respectively.

**Figure 4:**
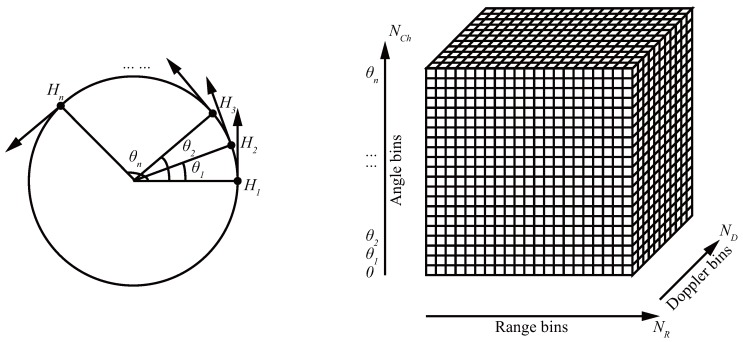
From projections to radar signal datacube.

The fast-time sampling frequency of the radar determines the number of range bins, and the pulse repetition frequency (i.e., slow-time sampling frequency) determines the Doppler bins. Radar received signals are forming a time series 1D signal after the analog-to-digital converter (ADC). In Figure 4, the fast-slow-time samples accumulated at each angle $\theta_{1},\;\theta_{2},\;...,\;\theta_{n}$ are reshaped by the number of range bins, $N_{R}$, and the number of Doppler bins, $N_{D}$. Hence, the sampling sequence of fast-time, slow-time and frames (angles) data are organized as a $N_{R}\times N_{D}\times N_{Ch}$ complex time domain data matrix. In our MMWCSAR system, the acquisition data format is the fast-time-slow-time-angle datacube, as the fast-time and slow-time data are associated with range and Doppler (projections), respectively. This datacube can be processed into range-Doppler angle data after pulse-Doppler processing. The IRT is related to the Doppler angle planar data for each range bin. Because the relative velocities of targets caused by circular movement project different velocities based on the azimuth/elevation location onto different angle profiles, the IRT method can be applied to reconstruct the image in each range bin of our imaging geometry, which is similar to computed tomography [15]. Some IRT-applied radar techniques can be found in [34]. Consequently, the Doppler-angle data matrix can be extracted for imaging restoration of each range bin. 3D imaging can be obtained from recovering 2D images for each range bin using the “2D + 1D” model [22].

For LFM waveform, its pulse-Doppler processing is coupled together [39]. However, in our approach, we implement compressed sensing (in Section 4) to improve the final image quality of the proposed radar. The pulse compression is done separately from the Doppler compression.

After forming a 3D datacube, the uncompressed received echo signals are pulse compressed by discrete Fourier transform (DFT) of the transmitted signal along the range axis. In Section 3, we separate the range profile and process the signal on 2D profile of the datacube to obtain the FOV figure on each range bin.

From the acquisition stage, we are using the fast-time, slow-time and angles in forming a 3D datacube. The pulse compression converts the datacube into the range-slow-time-angle datacube; and applying IRT to obtain range-azimuth-elevation datacube. The last datacube is the volume FOV figure of the actual image.

### 2.4. MMWCSAR Resolution

The MMWCSAR system has 3D imaging capacity. Therefore, the resolution is an essential topic for high resolution imaging.

For the range resolution, the range profile is independent of the Doppler and angle profiles. Thus the range resolution is:

(9) $$\Delta R=\frac{c}{2(BW)}\;.$$

For azimuth and elevation resolution, they are dependent on Doppler and angle profiles. Both resolutions are equivalent because the MMWCSAR is moving along a circular track with even angle spaces. Due to polar to Cartesian interpolation, the resolution of azimuth or elevation is higher around the rotation center and is lower at the edge. The azimuth/elevation resolution Δl is defined as:

(10) $$\Delta l=2l\sin\frac{\pi \left(PRI\right)N_{D}}{\omega }\;,$$

where *l* is the projection distance from the center. *l* has the limit from the center of origin to the azimuth/elevation edge, which is:

(11) $$0⩽l⩽R\tan\frac{\theta_{FOV}}{2}+r\;,$$

where *R* means the range at which we measure the azimuth/elevation resolution and $\theta_{FOV}$ denotes the FOV angle of radar looking vision. Because the resolution depends on the location of the azimuth/elevation FOV figure, in general, the worst resolution in a azimuth/elevation FOV figure is used to judge the MMWCSAR system’s azimuth/elevation resolution. Therefore, the resolution for the azimuth/elevation is:

(12) $$\Delta l=2\left(R\tan\frac{\theta_{FOV}}{2}+r\right)\sin\frac{\pi \left(PRI\right)N_{D}}{\omega }\;.$$

Note that the resolution of azimuth/elevation is dependent on the range at which we measure the azimuth/elevation resolution.

### 2.5. Constraints of Parameters

In order to reconstruct a high quality image, more data should be acquired in the 3D datacube in the acquisition stage. However, the number of data have some limitations as below.

#### 2.5.1. Constraints of Number of Doppler Bins

Because the radar is moving and targets are static within the sampling period, targets have relative velocities with respect to the MMWCSAR depending on their azimuth and elevation locations. If the target is far from the center of its range bin, the relative velocity increases. Accumulated Doppler bins define the resolution of the Doppler frequency based on targets’ azimuth and elevation locations. Hence, to better reflect the relative velocities of targets in the datacube, more Doppler bins are needed. We need to have enough Doppler bins to cover all of the relative velocities within the FOV angle:

(13) $$\frac{N_{D}+1}{2}\Delta v_{D}⩾\frac{4\pi r}{\omega }\sin\frac{\theta_{FOV}}{2}\;,$$

where $\Delta v_{D}$ is the velocity interval between two adjacent Doppler bins. $\Delta v_{D}$ is defined as:

(14) $$\Delta v_{D}=\frac{c}{2f_{c}\left(PRI\right)\left(N_{D}\right)}\;,$$

where *c* is the speed of the electromagnetic wave.

By choosing the appropriate number of Doppler bins, the system is able to capture all needed data for range-Doppler response within a limited given time period. This allows the system to capture enough frame data to form a range-Doppler-frames datacube. In this case, the frame data serve as the rotation angle. Therefore, the 3D datacube is consisting of range-Doppler-angle with the magnitude of targets. PRI also has a limit, which needs to cover all of the relative velocities with respect to radar to avoid blind speed:

(15) $$\frac{2\pi r}{\omega }⩽\frac{c}{2f_{c}\left(PRI\right)}\;.$$

#### 2.5.2. Constraints of the Number of Angle Bins

However, choosing too many Doppler bins results in fewer range-Doppler responses per full-round scan. To increase the number of angle bins, one needs to decrease the time used for capturing fast-time-slow-time samples. Hence, the system needs to take less time in capturing per fast-time-slow-time samples, so that allows more angle data (frames data) recorded through rotating. The sampling frame time for each fast-time-slow-time sample is:

(16) $$T_{Ch}=\left(PRI\right)\left(N_{D}\right)\;.$$

The radar movement is relatively constant when collecting fast-time-slow-time samples. That is to say, to achieve each sample within $10^{\circ }$ rotation, thus allowing 36 angle samples per full-round scan, we have another constraint:

(17) $$\frac{2\pi T_{Ch}}{\omega }⩽2\pi \left(\frac{10^{\circ }}{360^{\circ }}\right)\;.$$

Simplify the equation, and we get:

(18) $$\omega ⩾36\left(PRI\right)\left(N_{D}\right)\;.$$

The maximum detectable Doppler frequency is restricted by Doppler compression:

(19) $$f_{d,\max}=\frac{2\pi r}{\omega }\frac{2f_{c}}{c}⩽\frac{2}{PRI}\;.$$

The same, the minimum detectable Doppler frequency is restricted by:

(20) $$f_{d,\min}=\frac{2\pi r}{\omega }\frac{2f_{c}}{c}\frac{1}{N_{D}}⩽\frac{2}{\left(PRI\right)\left(N_{D}\right)}\;.$$

Merging Equations (13)–(20), we get constraints with our system. A proper choice of parameters is:

$$\begin{array}{c} N_{D}=25,\;PRI=30\times 10^{-6}\;s,\\ r=0.2\;m,\;\omega =0.6\;s/\mathrm{round}\;. \end{array}$$

In the later sections, simulation and experiment restrictions follow constraints discussed in this section. The different chosen parameters can result in different scanning schemes. Thus, this depends on targets and detecting scenarios. For example, we want to see metal objects concealed behind people’s clothes [40]. We need to increase the swinging rate and improve the FOV resolution. Using our constraints, we reduce our frames and PRI in order to meet the criteria.

## 3. Radar Imaging Processing

In this section, we discuss range calibration, as well as the imaging processing for the receiving datacube.

### 3.1. Range Calibration

From Section 2.1, additional range calibration is needed. This is because: the fan-shaped range bin should be converted into a plane-shaped range bin; the range profile of the radar should match with the rotation position, as we assumed the rotation radius is zero. From Figure 2, for a single target *A*, the displacement vector from radar is the actual measured range. The assumed range is from the origin, $\vec{OA}=\left(R_{1}\sin\beta_{1}\cos\alpha_{1},R_{1}\sin\beta_{1}\sin\alpha_{1},R_{1}\cos\beta_{1}\right)$, which is not changing throughout time. The radar location at different time is $\vec{OH}=\left(r\cos\left(2\pi t/\omega \right),0,r\sin\left(2\pi t/\omega \right)\right)$. Thus, the revised range is:

(21) $$\begin{array}{cc} R_{revised} & =\left|\vec{HA}\right|=\left|\vec{OA}-\vec{OH}\right|\\ & =\sqrt{{\left(R_{1}\sin\beta_{1}\cos\alpha_{1}-r\cos\frac{2\pi t}{\omega }\right)}^{2}+{\left(R_{1}\sin\beta_{1}\sin\alpha_{1}\right)}^{2}+{\left(R_{1}\cos\beta_{1}-r\sin\frac{2\pi t}{\omega }\right)}^{2}}\; \end{array}$$

The range difference of the revised range and assumed range is:

(22) $$\begin{array}{cc} R_{diff} & =R_{revised}-R_{assumed}=\left|\vec{HA}\right|-\left|\vec{OA}\right|\\ & =\sqrt{{\left(R_{1}\sin\beta_{1}\cos\alpha_{1}-r\cos\frac{2\pi t}{\omega }\right)}^{2}+{\left(R_{1}\sin\beta_{1}\sin\alpha_{1}\right)}^{2}+{\left(R_{1}\cos\beta_{1}-r\sin\frac{2\pi t}{\omega }\right)}^{2}}\\ & \;-\sqrt{{\left(R_{1}\sin\beta_{1}\cos\alpha_{1}\right)}^{2}+{\left(R_{1}\sin\beta_{1}\sin\alpha_{1}\right)}^{2}+{\left(R_{1}\cos\beta_{1}\right)}^{2}}\; \end{array}$$

As $\left|\vec{HA}\right|$ is only dependent on the time of the rotation, thus the range-Doppler-angle 3D datacube requires the calibration of range using $R_{diff}$ at each range-Doppler planar data corresponding to time. Time in our MMWCSAR system is related to the rotation angle θ, which is the angle profile of the datacube. A time-indexed matrix $V^{-1}$ is thus able to perform the range calibration in compressed sensing in Section 4.

### 3.2. Radon Transform and 3D Imaging Reconstruction

Radon transform and 3D image reconstruction are illustrated in many applications, i.e., computed tomography [15] and thermoacoustic tomography [41]. Obtaining tomographic image from projections data and conversion has been introduced in [15,18,19,20,42,43]. The cross-sectional image of targets at each range bin consists of projection angle θ and the projection distance ξ. The IRT is performed for reconstruction tomography.

#### 3.2.1. Radon Transform and Inverse Radon Transform

The Radon transform is an integral transform converts the 2D image to its projections, p(ξ,θ). The Radon transform can be defined as [15]:

(23) $$p\left(\xi ,\theta \right)=\int_{-\infty }^{\infty }\int_{-\infty }^{\infty }f\left(x,y\right)\delta \left(\xi -x\cos\theta -y\sin\theta \right)dxdy\;,$$

in which f(x,y) denotes the original 2D density distribution function indexed by *x* and *y*; ξ is the projection distance from center; θ is the projection angle; and δ(·) is the Dirac delta function.

Its inverse transform, IRT, is widely used for image reconstructions. IRT can reconstruct the image from the projection data by several techniques. Traditionally, people used the back-projection theorem to recover the inverse Radon transform [42]. A recent CT development inspired the central slice theorem (CST) [15], which is the simplest method conceptually compared to back-projection and iterative algebraic techniques [20]. The theorem states that the 2D Fourier transform (FT) of the original function f(x,y) is the function of 1D Fourier transforms of the projection slices in the order of angles. The 2D FT of the original function is:

(24) $$F\left(u,v\right)=\int_{-\infty }^{\infty }\int_{-\infty }^{\infty }f\left(x,y\right)\exp\left[-j2\pi \left(ux+vy\right)\right]dxdy\;.$$

P(ρ,θ) is the 1D FT series of the projections p(ξ,θ), which can be represent by:

(25) $$P\left(\rho ,\theta \right)=\int_{-\infty }^{\infty }p\left(\xi ,\theta \right)exp\left(-j2\pi \rho \xi \right)d\xi \;.$$

Hence, the 2D Fourier domain function F(u,v) can be obtained from the Fourier domain function P(ρ,θ) by interpolation between polar and Cartesian coordinates:

(26) $${P\left(\rho ,\theta \right)=F\left(u,v\right)|}_{u=\rho \cos\theta ,v=\rho \sin\theta }\;.$$

Therefore, the spatial image can be obtained from projection slices by CST. In this MMWCSAR system, the Doppler-angle data can be converted by this algorithm to reconstruct FOV images of targets’ scene at different range bins.

#### 3.2.2. 3D Imaging Reconstruction and Point Spread Function

The profile of range data is independent of the Doppler-angle data after the geometric calibration of the range. The Doppler-angle planar data can be used to reconstruct the 3D image. For each range bin, Doppler-angle data are the projections of the 2D azimuth/elevation FOV figure in the back-projection domain [44]. The transformation of the Doppler-angle and 2D FOV figure is basically using the Radon-inverse Radon transform pair. This pair can be expressed as an invertible matrix of the Radon transform-IRT linear matrix with interpolation of the Cartesian and polar coordinates. Thus, by using the “2D + 1D” model [22], 2D azimuth-elevation FOV images are resolved at different range bins. These images are adding the range bin profile to reconstruct a volume FOV figure. This allows 3D imaging.

**Figure 5:**
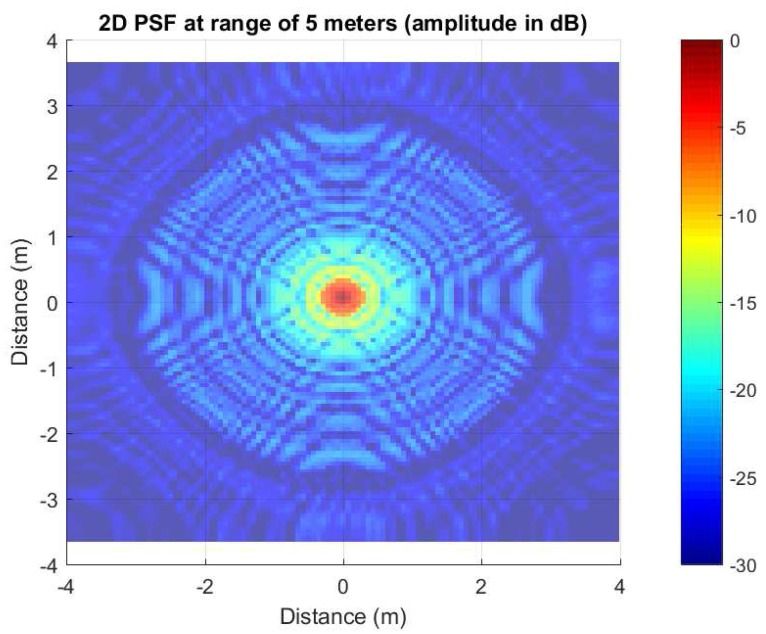
2D point spread function (PSF) for the point scatterer located at the center (0,0) at a range of 5 m (amplitude in dB).

For the point spread function (PSF), it defines the response of an imaging system to a point source [45]. The MMWCSAR system azimuth-elevation FOV image is formed as the response from point scatterers with the following summation:

(27) $$MMWCSAR\left(x,y\right)=\sum_{i=1}^{N}A_{i}\delta \left(x-x_{i},y-y_{i}\right)*h\left(x,y\right)\;,$$

where *N* represents the total number of point scatterers and $A_{i}$ is the scattered field amplitude. x,y is the azimuth and elevation location, respectively. h(x,y) is the PSF of the MMWCSAR system. h(x,y) can be regarded as the system’s impulse response to any point scatterers of the target. The image can be expressed as the convolution of scatterers with the PSF. For our system, the PSF for one scatterer at a range of 5 m at the center of the radar view is shown in Figure 5.

From Figure 5, the side lobe level is −13 dB. The PSF has circular side lobes from the center. This is caused by IRT along all directions. The side lobes exhibit a sinc-function shape because of the finite bandwidth in the Doppler and angle domain. The common way to suppress the side lobes is to use windowing. In our MMWCSAR system experiment, we are using the Hanning window.

## 4. Compressed Sensing for 3D Imaging Radar System

In terms of improving sensing results and boosting the rotating accuracy, compressed sensing is used in our MMWCSAR.

### 4.1. Compressed Sensing Review

The basic CS idea is reviewed below. Suppose that we have a two-dimensional (2D) image with a size of A×B, which can be extract into a one-dimensional (1D) vector $\vec{f}$ with a length of N=AB×1. Any 1D vectors can be constructed by sampling the sensing basis $\phi =[\phi_{1}|\phi_{2}|...|\phi_{M}]$ [23]. *M* is the number of measurements of the sensing basis ϕ and is smaller than the length of $\vec{f}$. Thus, the sampling vector $\vec{y}$ can be expressed as:

(28) $$\vec{y}=\phi_{k}\vec{f},\;k=1,2,...,M\;.$$

The restriction laying the *K*-sparse signal $\vec{f}$ should have K<M<N, which allows the signal recovery from the *M* measurements.

CS requires the sparsity and incoherent sampling. For sparsity, we have $\vec{f}\in R^{N}$, which is N=AB pixels in the 2D image. For an orthogonal transform, i.e., discrete cosine transform (DCT), almost all of the pixels could have sparse expansion without much perceptual loss. This results in a representation basis $\psi =[\psi_{1}|\psi_{2}|...|\psi_{N}]$ [23], which allows the following representation:

(29) $$\vec{f}=\sum_{i=1}^{N}\psi_{i}x_{i}\;.$$

For incoherence sampling, from [23], the required sensing basis and representation basis should have the following incoherence parameter:

(30) $$u\left(\phi ,\psi \right)=\sqrt{n}\max_{1⩽k,j⩽n}\left|\langle \phi_{k},\psi_{j}\rangle \right|\;.$$

The reconstruction uses $l_{1}$-norm minimization [46,47,48]. The proposed solution $\hat{f}$ is constructed by $\hat{f}=\phi \hat{x}$, where $\hat{x}$ is the solution to the convex optimization:

(31) $$\hat{x}=\min\left\{{∥\vec{x}∥}_{1}:\vec{y}=\phi \psi \vec{x}\right\}\;.$$

Thus, the measurement $\hat{x}$ is produced by the sparsest signal of the decoding model.

### 4.2. Compressed Sensing on Doppler-Angle Data

As the Doppler profile is in the frequency domain and the angle profile is in the spatial domain, slow-time data are used, and the pulse compression is done apart from pulse-Doppler compression. Consequently, we have a DFT matrix of $U^{-1}$. $U^{-1}$ is a 2D complex matrix, which has 1D DFT along fast-time bins, with slow-time and angle profiles repeating to match the whole 3D data size. U is then the transform from range frequency to fast-time samples with repetition of slow-time and angle profiles. The range calibration matrix can be expressed as $V^{-1}$.

We also implement the RT and IRT matrix for the CS. The projections on each range profile are the projection domain data of the 2D azimuth-elevation FOV figure. We use a rectangular IRT matrix $W^{-1}$ transform the slow-time-angle 2D data to the 2D azimuth-elevation FOV figure with the range profile repeating to match the whole 3D data size. The IRT matrix representation $W^{-1}$ is produced combined with linear interpolation from polar coordinates to Cartesian coordinates. Thus, we have a conversion from the original 1D-reshaped range-slow-time-angle data $\vec{b}$ to the 1D-reshaped calibrated range-azimuth-elevation data $\vec{x}$:

(32) $$\vec{b}=\mathrm{UVW}\vec{x}\;.$$

Fourier transform and Radon transform are linear transforms. Both of the transformation matrices are invertible. In addition, both $U^{-1}$ and $W^{-1}$ work on 1D-reshaped range-slow-time-angle data. Hence, CS requires solving $\vec{x}$ by using three linear mapping matrices $W^{-1}V^{-1}U^{-1}$:

(33) $$\vec{x}=W^{-1}V^{-1}U^{-1}\vec{b}\;.$$

In this approach, the representation basis ψ is represented as:

(34) ψ=UVW.

For any condensed signal $\vec{y}$, the sensing of the range-slow-time-angle data $\vec{b}$ can be expressed as:

(35) $$\vec{y}=\phi \vec{b}\;.$$

Therefore, the reconstruction is able to be implemented by using $l_{1}$-minimization on the sensing basis ϕ and the representation basis ψ from Equation (34):

(36) $$\hat{x}=\min\left\{{∥\vec{x}∥}_{1}:\vec{y}=\phi \mathrm{UVW}\vec{x}\right\}\;.$$

To summarize, $U^{-1}$ converts the 1D-reshaped fast-time-slow-time-angle original data into 1D-reshaped range-slow-time-angle data. $V^{-1}$ is the calibration matrix of the range bins. $W^{-1}$ transforms the range-slow-time-angle data into 1D-reshaped range-azimuth-elevation FOV figure.

In our simulation and experiment, we solve the convex optimization through the MATLAB primal-dual interior point method [49,50]. The sparsity of Doppler-angle planar data is exploited. Because angle data are limited due to PRI and the ADC sampling rate when MMWCSAR accumulates data, the Doppler-angle data form a sparsity signal (multiple sinusoidal shapes) from targets. Through the optimization, the sparsity of the original slow-time-angle signal can be exploited to recover from fewer samples than that of the Nyquist sampling theorem [23]. Therefore, the compressed sensing is able to be implemented to recover the 3D imaging from fast-time-slow-time-angle datacube.

### 4.3. Sensing Basis ϕ Selection

We first define the compressed sensing ratio (CSR) as $R_{CS}$, expressed as:

(37) $$R_{CS}=\frac{N}{M}\;,$$

*M* and *N* are the number of rows and columns of the sensing basis, respectively. CSR is the ratio of the length of the expected signal $\vec{b}$ over the length of condensed signal $\vec{y}$. It specifies the reconstruction quality and size of the sensing basis and representation basis. The slow-time-angle data CS is done with compressing both slow-time and angle. These data are transformed on each calibrated range profile. Sensing basis ϕ can be expressed as follows:

(1) Reduced rotation acquisition matrix:
  The matrix is expressed as:
  (38) $$\phi_{k+h,k}=\left\{\begin{array}{cc} 1, & k=1,2,...,M\;\&\;0⩽k+h⩽M\\ 0, & \mathrm{otherwise} \end{array}\right.\;.$$
  *h* is the offset depends on the rotation span. This allows sensing the expected signal with reduced rotation angles. The sensing basis reconstructs the condensed signal into a full angular projections Doppler-angle signal along the angle profile. Different projections provide different IRT responses. Thus, this sensing basis enables the sampling at fewer angle bins, which reduces the swinging inaccuracy. This sensing matrix is also applicable to the Doppler profile, which also improves projections along angle profile.
(2) Reduced sampling matrix:
  This matrix is expressed as:
  (39) $$\phi_{k,\left\lfloor R_{CS}\right\rfloor \left(k-1\right)+1}=\left\{\begin{array}{cc} 1, & k=1,2,...,M\\ 0, & \mathrm{otherwise} \end{array}\right.\;.$$
  $\left\lfloor R_{CS}\right\rfloor$ denotes the max integer smaller than $R_{CS}$. This allows the sensing basis sensing the expected signal at its higher sampling rate. The sensing basis converts the condensed signal with more projection data along the Doppler or angle bins. This method avoids swinging at an inconsistent rate of the velocity. This allows the signal recovery at better constant sinusoidal-shaped Doppler-angle data.
(3) Gaussian or random matrix:
  The Gaussian sensing basis is shown below:
  (40) $$\phi_{k}=\exp\left[-{\left(k-\mu \right)}^{2}/\left(2\sigma^{2}\right)\right],\;k=1,2,...,M\;.$$
  μ is the expected value of the Gaussian, and σ is the standard variance of the Gaussian. The random matrix is also applicable. This method allows the sampling followed the normal compressed sensing procedure. This corresponds to some CS applications in MRI, i.e., [26]. The sensing basis converts the condensed signal in a smoother way. It gives the compensation to the signal. It allows the signal recovery with much more precise points along the Doppler-angle data.

The following simulation and experiment will provide the results of CS involved in the MMWCSAR system. The comparison will be provided with the IRT method.

## 5. Simulation and Experiment

### 5.1. Simulation Setup and Results

MMWCSAR is able to reconstruct the 3D image along each range bin. The following simulation provides a FOV figure of range of 5 m. The MMWCSAR system parameters can be found in Table 1. The four targets’ scheme parameters are shown in Table 2.

As the MMWCSAR system is producing the radar transmitted LFM waveforms, the received signal together with produced Gaussian noise is analyzed with the IRT method and the involved CS method. Additionally, in order to process Doppler-angle data without the influence of the range profile, the range calibration is necessary in the simulation. From the 3D datacube, the 3D fan-shaped range bin is accumulated. From Equation (22), we process every Doppler-angle slice along the range axis with a calibration matrix V to convert the fan-shaped range bin into a plane-shaped range bin.

**Figure 6:**
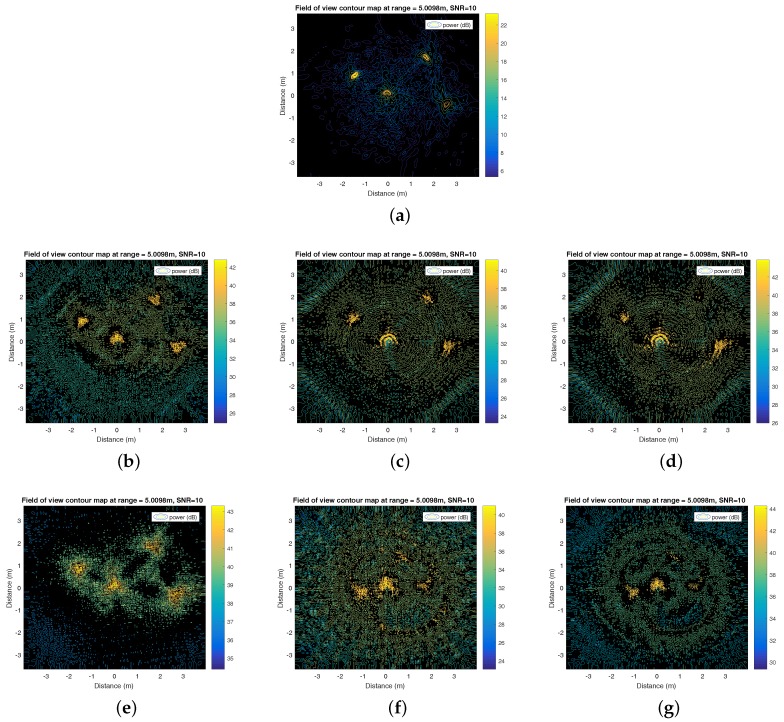
Simulation results (front view from radar panel at 5 m, the *x* axis and *y* axis are azimuth and elevation distance from the center, respectively). (**a**) Inverse Radon transform (IRT) method with the perfect setup; (**b**) compressed sensing (CS) applying Equation (38) to the angle profile with $R_{CS}=1/2$; (**c**) CS applying Equation (39) to the angle profile with $R_{CS}=1/2$; (**d**) CS applying Equation (40) to the angle profile with $R_{CS}=1/2$; (**e**) CS applying Equation (38) to the slow-time profile with $R_{CS}=1/2$; (**f**) CS applying Equation (39) to the slow-time profile with $R_{CS}=1/2$; (**g**) CS applying Equation (40) to the slow-time profile with $R_{CS}=1/2$.

The simulation results are shown in Figure 6 with FOV figures of 5 m seen from the radar panel. Figure 6a addresses the IRT method with the perfect setup without swinging inaccuracies. The velocity of the radar is changing evenly with direction and a constant magnitude. Figure 6b–g introduces all of the involved CS method by applying different sensing basis on slow-time-angle bins separately. For the experiment, we will use the Equation (38) matrix for the slow-time profile as in Figure 6d, as it provides high contrast and better scatterer indication.

### 5.2. Experiment Setup and Results

**Figure 7:**
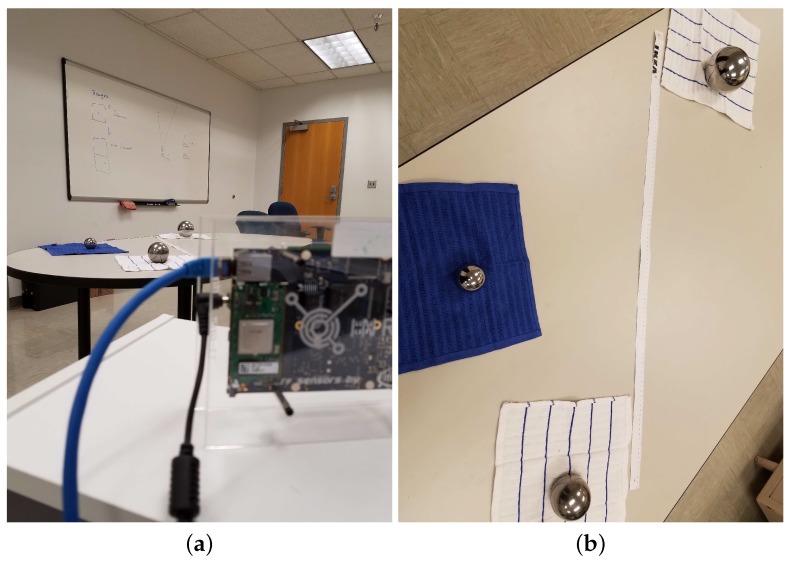
Experiment setup. (**a**) Radar position with antenna facing three ball targets; (**b**) three ball targets’ lineup.

With the equipment from the INRAS MIMO radar [51,52] and three metal ball targets, a basic system can be set up. The INRAS MIMO radar is a four-transmitter, eight-receiver MIMO radar. We use only one transceiver element, which satisfies the MMWCSAR configuration. The radar works at 77 GHz in range-Doppler mode. The ball targets are made from metal with a high RCS, which can produce high reflective beams compared to other targets’ reflections. The parameters for the experiment can be seen in Table 3. The experimental setup scheme is shown in Figure 7a. The ball lineup with measurement is given in Figure 7b. The balls are set at a range of 1.21 m, 1.66 m and 2.16 m from the radar panel, respectively.

The rotating angle per frame is around 15.3°, based on the calculation of $360^{\circ }$ over the number of frames $N_{Ch}$ accumulated by the signal processing unit. Multiple swinging circles are recorded in order to reduce the impact of inaccuracies caused by hand. Additionally, in order to minimize the hand swinging inaccuracy on Doppler-angle data, the range calibration is necessary in the experiment. Otherwise, some ghost targets will be observed around detected targets. The calibration method is similar to that of the simulation part.

**Figure 8:**
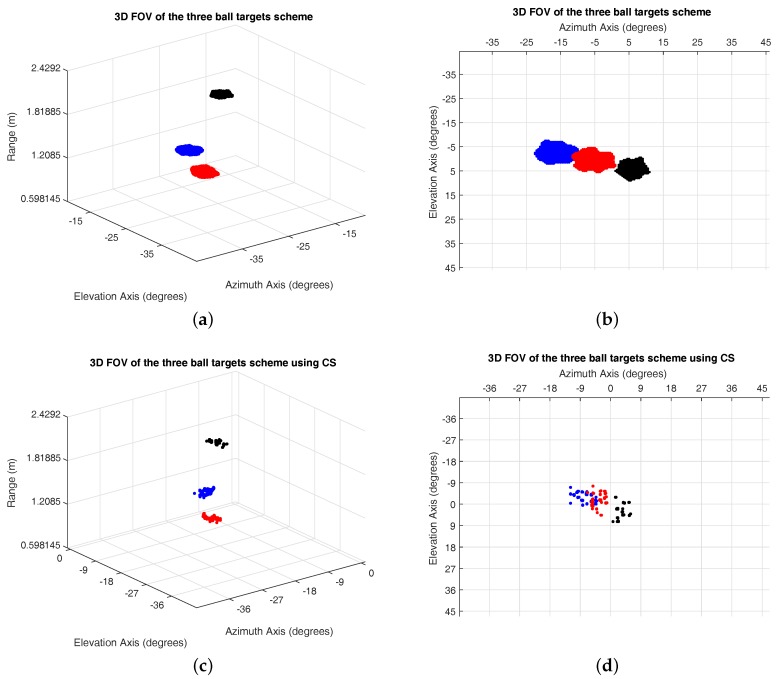
Experiment results. (**a**,**b**) IRT method; (**c**,**d**) CS applying Equation (38) to the slow-time profile.

The results provide a 3D scattering plot of the FOV in front of the MMWCSAR. The indices of the figure are the range in meters, the azimuth angle and the elevation angle in degrees. The figures of the applying IRT and CS methods can be seen in Figure 8. We add the threshold to distinguish the metal ball targets from the table, the board and walls. The metal ball targets’ reconstruction using the IRT method (Figure 8a,b) gives a slightly larger size in azimuth and elevation directions compared to the actual size. Reconstruction using the CS method (Figure 8c,d) provides a more precise scatterer indication, but lost the shape of the metal ball. The range resolution is 37.5 mm for both methods, as the range resolution is dependent on bandwidth only. The azimuth/elevation resolution based on Equation (12) is 36.2 mm for the 2.16-m range bin. The experiment measured azimuth/elevation resolution is 44 mm from the worst recognized 2.16-m target. The CS method provides a better scatterer indication, but the IRT method gives a more precise ball shape. In conclusion, the MMWCSAR system using both methods is capable of 3D imaging by moving the radar along a circular track.

## 6. Discussion

### 6.1. Millimeter Wave 3D Imaging Radar

For both the IRT and CS method, the 3D range-azimuth-elevation FOV figure is recovered. The proposed system can have the following advantages:

• Fast:

To recover the 3D imaging, the proposed system needs to collect data when the system is moving. A full circular movement track is needed for the IRT method to collect data, while the CS method can greatly decrease the number of samples. It takes only seconds for MMWCSAR to recover an FOV figure.

• Portable:

Due to its size, the proposed system can be wearable for 3D imaging. Both the IRT and CS methods produce convincing results from the simulation and experiment.

• High resolution:

With other SAR imaging device shown in [21,22], the resolution is around 0.1 m. Our MMWCSAR working on 77 GHz can achieve 37.5-mm range resolution and 36.2-mm azimuth/elevation resolution at a range of 2.16 m (from Equation (12) and experiment data), theoretically. Due to hand swinging inaccuracy and noise, the resolution is reduced to around 44 mm in experimental measurements.

Due to limited access to resources, we rotate the platform manually and record the time we finish one rotation. Due to the rough estimation of the rotation, the accuracy and resolution are lower than the theoretical analysis. To compensate the errors, we set a calibration target, e.g., we use a corner reflector at a range bin without any other objects; we track the errors reading the Doppler-angle profile of the corner reflector. After compensation, the imaging still has decreased resolution. However, if we can integrate an inertial sensor into the system to extract real-time velocity information, a more precise and accurate figure could come out.

### 6.2. Comparison with the Radon Method and the Compressed Sensing Method

The conversion of the 3D data matrix is linear, and the signal is sparse. The inverse transform of the matrix is accessible, thus allowing the involved CS method. Comparing to the IRT method, the CS method gives the following advantages:

• Flexible:

From the simulation and experiment, it is clear that the involved CS MMWCSAR system is more flexible on data reconstruction. The calculations in the signal processing module are only involved in the matrix multiplexing and solving $l_{1}$-minimization. The acquisition is allowed more freedom. Based on the sampling of targets, the CS method is able to reconstruct a better image than that of the IRT method. Data accumulated are not limited to a full circle. Mistakes and errors can be eliminated from the CS method, as well. Besides, $R_{CS}=1/2$ is used for both simulation and experiment. It is flexible to adjust the compressed sensing ratio to improve the MMWCSAR FOV image further.

• High-SNR:

The peak of targets is more recognizable with a high decibel difference to the background compared to that of the IRT method. For example, in the simulation part, the CS provides a 43 dB peak identification to a 24 dB peak in the IRT method. The peak power is measured as −32.6 dB in the CS method compared to −80.8 dB in the IRT method. Besides, improvements on scatterer indication are also noticeable. In the experiment part, targets’ reflective scatterers are shown in more accurate locations. Targets are more easily recognized by the involved CS method of MMWCSAR.

• Fast-acquisition:

As matrix transformation is implemented in the CS method. Huge data convolution along different axes is accomplished. Fast implementation on acquisition is achieved at the cost of more time spent on signal processing. It is an advanced signal processing method used in large data matrices in modern imaging devices.

## 7. Conclusions

In this paper, we present a radar system named MMWCSAR. It can generate a high resolution 3D image. The resolution and constraints of the MMWCSAR platform are discussed. We take a step further with the signal processing module of our system. We discussed the range calibration and 3D imaging reconstruction. In addition to using the IRT method, the involved CS matrix transformation of the original data to the final volume FOV image is shown.

The proposed radar system is efficient, portable and fast for widespread use. The radar transceiver used in this design is more affordable than using an MIMO imaging or traditional SAR imaging. It avoids the large antenna, as well as the complex radar transceivers. A user just needs to move the radar along a circular track. A high resolution volume FOV figure can be extracted using our algorithms.

## Figures and Tables

**Table 1:** MMWCSAR simulation setup. PRI, pulse repetition interval.

Parameters	Symbol	Value
Center frequency	$f_{c}$	76.5 GHz
Chirp starting frequency	$f_{start}$	76 GHz
Chirp end frequency	$f_{stop}$	77 GHz
PRI	PRI	30 × 10${}^{-6}$ s
Chirp duration	$T_{P}$	20 × 10${}^{-6}$ s
Fast time samples	$N_{R}$	400
Slow time samples	$N_{D}$	100
Frame samples	$N_{Ch}$	68
Sampling frequency	$f_{s}$	20 × 10${}^{6}$ Hz
Angular velocity	ω	0.2 s/round
Rotation radius	*r*	0.6 m
Signal to noise ratio (SNR)	SNR	10 dB

**Table 2:** Four targets’ scheme setup. RCS, radar cross-section.

Parameters	Target 1	Target 2	Target 3	Target 4
Range	5 m	5 m	5 m	5 m
Azimuth location	−1.2941 m	1.7101 m	0 m	2.1131 m
Elevation location	0.8682 m	1.7101 m	0 m	−0.4358 m
RCS	1 m${}^{2}$	1 m${}^{2}$	1 m${}^{2}$	1 m${}^{2}$

**Table 3:** Three ball targets’ scheme setup.

Parameters	Target 1	Target 2	Target 3
Range	1.21 m	1.66 m	2.16 m
Azimuth angle α	≈−2${}^{\circ }$	≈−5${}^{\circ }$	≈5${}^{\circ }$
Elevation angle β	≈0${}^{\circ }$	≈0${}^{\circ }$	≈0${}^{\circ }$
